# Structure–function engineering of novel fish gelatin-derived multifunctional peptides using high-resolution peptidomics and bioinformatics

**DOI:** 10.1038/s41598-021-86808-9

**Published:** 2021-04-01

**Authors:** Armin Mirzapour-Kouhdasht, Marzieh Moosavi-Nasab, Chul Won Lee, Hyosuk Yun, Jong-Bang Eun

**Affiliations:** 1 Department of Food Science and Technology, School of Agriculture, Shiraz University, Shiraz, Iran; 2 Seafood Processing Research Group, School of Agriculture, Shiraz University, Shiraz, Iran; 3 Department of Chemistry, Chonnam National University, Gwangju, 61186 South Korea; 4 Department of Integrative Food, Bioscience and Biotechnology, Chonnam National University, Gwangju, South Korea

**Keywords:** Biochemistry, Biotechnology, Chemistry

## Abstract

The multifunctional properties of fish gelatin hydrolysates have not been completely elucidated. Here, the biological characterization of these peptides was performed to engineer multifunctional peptides. Bioactive peptides were produced from mackerel byproducts via successive enzymatic hydrolysis reactions using subtilisin A and actinidin as microbial and herbal proteases. The antibacterial activity against both gram-negative and -positive food-borne pathogens, including *Escherichia coli, Pseudomonas aeruginosa, Staphylococcus aureus*, and *Klebsiella pneumoniae*, as well as the inhibitory potential of angiotensin-converting enzyme (ACE) and dipeptidyl peptidase IV (DPP-IV), was accessed in vitro. The synthesized peptides demonstrated multifunctional properties, which were further confirmed by in silico protocols. The ACE and DPP-IV inhibitory (IC_50_) values of P1, P2, and P3 were 0.92 and 0.87, 0.51 and 0.93, 0.78 and 1.16 mg mL^−1^, respectively. Moreover, the binding energy was sufficient for all three peptides to inhibit both ACE and DPP-IV enzymes with excellent three-dimensional conformation (RMSD = 0.000) for all six docking mechanisms.

## Introduction

Gelatin is a hydrocolloid that can be produced by partial hydrolysis of collagen from different sources, including fish byproducts, which represents an economical or environmental advantage. Gelatin can be applied in food and pharmaceutical industries; however, bioactive peptides derived from this glycine and proline-rich protein^1,2^ can be more advantageous. Some di/tri/tetrapeptides with a proline at their C-terminus can suppress the dipeptidyl peptidase IV (DPP-IV) enzyme, which participates in the incretin hormone processing^3^ and can regulate diabetes mellitus type II^4,5^. Hence, some synthetic drugs use DPP-IV inhibitory activity for the treatment of diabetes type II, but with significant side effects such as hypoglycemia, weight gain, increased bowel movement frequency, diarrhea, nausea, and abdominal pain^6^. Therefore, new natural compounds with antidiabetic activity could represent a promising solution for these problems.

Angiotensin-converting enzyme (ACE) is a zinc protease that plays a major role in regulating human blood pressure by converting inactive angiotensin I (a decapeptide) into a potent vasoconstrictor agent called angiotensin II (an octapeptide) as well as by inactivating a depressor peptide called bradykinin^7^. The presence of specific amino acids such as proline at the N- or C-terminus of peptides can change their activity; however, some studies have described ACE inhibitory peptides without proline at the N-/C-terminus^8,9^.

Few studies have explored the antibacterial activity of gelatin-derived peptides, contrasting with several studies that have described their other bioactivities. The charge state, hydrophobicity, molecular weight attributes, and the amino acid composition and sequence of peptides have important effects on their antibacterial activity^10–14^. Thus, this study aimed to engineer multifunctional peptides derived from fish gelatin based on their structure–function characteristics using high resolution peptidomics and bioinformatics approaches. Specifically, the antidiabetic, antihypertensive, and antibacterial activities of the peptides were targeted.

## Methods

### Raw materials, bacterial strains, and reagents

The Barred mackerel (*Scomberomorus commerson*) and green kiwifruit (*Actinidia deliciosa*) were provided from a local market (Gwangju, South Korea). Bacterial strains were obtained from the American Type Culture Collection (ATCC, Manassas, VA, USA). Subtilisin A from *Bacillus licheniformis* (406.80 U mg^−1^) was purchased from the National Institute of Genetic Engineering and Biotechnology (Tehran, Iran). All chemicals and reagents were of analytical grade and obtained from Sigma-Aldrich (St. Louis, MO, USA).

### Gelatin extraction and hydrolysis

Gelatin was extracted from Mackerel byproducts as previously described^15^. Briefly, the byproducts were cut into 2 cm^2^ pieces. After soaking in a NaOH solution (0.1 N, pH 13) at a ratio of 1:5 (w/v) for 24 h (the solution was changed every 6 h) and washing with distilled water until neutral pH, defatting was performed using 10% n-butanol at a ratio of 1:5 (w/v). The gelatin was extract at 70.71 °C in distilled water for 5.85 h. Finally, the gelatin powder was obtained by filtering the solution using a Whatman No.1 filter paper, followed by lyophilization.

A gelatin solution of 2.5% (w/v) concentration was prepared in Tris-HCl buffer. The hydrolysis protocol was obtained from a previous study^16^. A combination of subtilisin A and actinidin (1:100, w/w) was used to hydrolyze the gelatin. First the subtilisin A reacted at 55 °C and pH 8.5 for 3 h, followed by the actinidin, which reacted at 37 °C and pH 7.5 for another 3 h. After centrifugation (8000 × *g* for 15 min), the supernatant was lyophilized as gelatin hydrolysates and kept at − 20 °C until further experiments.

### Degree of depolymerization (DP)

DP during 6 h of enzymatic reaction was determined using the TNBS method described by Adler-Nissen^17^. An aliquot of 250 µL of samples were prepared and mixed with 2 mL of 0.2125 M sodium phosphate buffer (pH 8.2). Subsequently, the 0.1% TNBS solution (2 mL) was added to the mixture and incubated at 50 °C for 1 h in a dark chamber. The reaction was stopped by adding 4 mL of 0.1 N HCl. The absorbance of the samples at 340 nm was detected after keeping them at ambient temperature for 30 min. A solution of 0.2 mM L-leucine was used as standard. The DP (%) was calculated as:

1 $${\text{DP}} \left( \% \right) = \left( {\frac{{{\text{AN}}_{2} { }{-}{\text{ AN}}_{1} }}{{{\text{N}}_{{{\text{pb}}}} }}} \right) \times 100$$

where AN_2_ and AN_1_ represent the amino nitrogen content (mg g^−1^) of the protein after and before hydrolysis reaction, respectively, and the N_pb_ represents the nitrogen content of the peptide bonds in the protein (mg g^−1^). The N_pb_ of 155.5 mg g^−1^ was used for fish gelatin^2^.

### Antibacterial (agar dilution) assay

The hydrolysates were investigated for their antibacterial activity against *E. coli* (ATCC 8739)*, P. aeruginosa* (ATCC 9027)*, S. aureus* (ATCC 6538)*,* and *K. pneumoniae* (ATCC 43816) using the agar dilution method^16^. Briefly, 50 μL of different concentrations of peptides were prepared in sterile Mueller Hinton broth. A volume of 30 μL of resazurin indicator solution (0.02%) was added to each well. An equal volume of bacterial suspension (0.5 McFarland standard) and sterile Mueller Hinton broth was then added to the well and incubated at 37 °C for 24 h. Sterile Mueller Hinton broth and 20 μg mL^−1^ gentamycin were used as blank and positive controls, respectively. Afterward, the minimum inhibitory concentration (MIC) was determined as the lowest concentration of the peptides at which no visible growth was observed, compared to the positive control. All dilutions were performed in duplicate.

### In vitro* antihypertensive assay*

A spectrophotometrical method was used to determine the antihypertensive activity of peptides according to the procedure of Asoodeh, et al.^18^, with some modifications. ACE (22 µL at 50 mU mL^−1^) was mixed with 50 µL of the peptides at increasing concentrations (0.1, 0.2, 0.4, 0.8, 1, and 2 mg mL^−1^). FAPGG (100 µL at 0.5 mM) and 150 µL of ACE buffer (50 mM Tris-HCl [pH 7.5] containing 0.3 M NaCl and 1 mM ZnCl2) were added to the mixture, followed by monitoring at 340 nm for 60 min. The antihypertensive activity was calculated as follows:

2 $$\mathrm{Antihypertensive}\mathrm{activity} \left(\%\right)=\left(1-\frac{{\mathrm{\Delta Abs}}_{sample}}{{\mathrm{\Delta Abs}}_{control}}\right)\times 100$$

The ACE buffer was added to the mixture instead of the peptides for control. For determination of the experiment precision, captopril (15 nM) was used as positive control. The half maximal inhibitory concentration (IC_50_) was defined as the concentration of the peptides at which 50% of ACE activity was inhibited. All experiments were performed in triplicate.

### In vitro* antidiabetic assay*

To assess antidiabetic activity, a pre-incubated solution (50 μL) containing 30 μL Tris-HCl buffer, 5 μL of peptides (0.1, 0.2, 0.4, 0.8, 1, 2, and 4 mg mL^−1^) or standard (5 μM), and 15 μL glycine-proline-proline-p-niroanilide were poured into the wells of a 96-well microplate. Afterward, 50 μL DPP-IV (0.02 U mL^−1^) was added to the mixture and incubated at 37 °C for 30 min. The reaction was stopped by adding 100 μL sodium acetate (1 M, pH 4). Absorbance at 405 nm was measured in a microplate reader. Diprotein-A (isoleucine-proline-isoleucine) was used as a standard DPP-IV inhibitor. The antidiabetic activity was calculated as follows:

3 $${\text{DPP - IV}}\;{\text{inhibitory}}\;{\text{activity}}(\% ) = \left( {1 - \frac{{{\text{A}}_{sample} - {\text{A}}_{blanck} }}{{{\text{A}}_{positice control} - {\text{A}}_{negative control} }}} \right) \times 100$$

Tris-HCl (100 mM) was added to the mixture instead of DPP-IV, peptide, or peptide plus DPP-IV to represent a blank, positive, and negative control, respectively^19^.

### Peptidomics approach

#### Ultrafiltration of hydrolysates

Gelatin hydrolysates were passed through 30, 10, and 3 kDa ultrafiltration amicon tubes at 4 °C. The resultant fractions were tested for antibacterial, antihypertensive, and antidiabetic activities to select the most active fraction for the next experiment.

#### Ion exchange chromatography

The fraction with the highest biological activity obtained from the above-described protocol was further purified using an AKTA prime plus (GE healthcare, Chicago, IL, USA) machine equipped with a HiTrap Q HP (GE healthcare) column. Buffer A composed of 20 mM NH_4_HCO_3_ (pH 8.3) containing 10 mM NaCl and buffer B composed of 20 mM NH_4_HCO_3_ (pH 8.3) containing 1 M NaCl were used as eluents. The gradient of 1–100% with a ratio of 1% per minute was applied and detected at 220 nm. Fractions of 3 mL were collected and stored at − 20 °C for further experiments.

#### Solid phase extraction (SPE)

Fractions with the highest activity obtained by ion exchange chromatography were pooled and further purified and desalted by solid phase extraction method using a Sep-Pak Plus C18 cartridge (Waters Corporation, Milford, MA, USA) with a volume of 1 mL. Water soluble peptides were eluted using 1 mL of high-performance liquid chromatography (HPLC) grade H_2_O. The remainder of the peptides were sequentially eluted using 1 mL of 5%, 10%, 15%, 20%, 25%, and 30% (v/v) ACN. The resultant fractions were pooled and lyophilized. Fractions of 10 mL collected and analyzed by an analytical reverse phase (RP)-HPLC Shimadzu 10AD system (Tokyo, Japan) equipped with a SunFire C18 column (4.6 × 250 mm; Waters Corporation). Buffer A (100% H_2_O containing 0.05% TFA) and buffer B (100% ACN containing 0.05% TFA) were applied with a gradient of 5–35%. The peaks were detected at 220 nm.

#### RP-HPLC

Further purification was conducted using a preparative RP-HPLC system equipped with a XBridge Prep C18 column (19 × 250 mm; Waters Corporation). Prior to analysis, the sample was dissolved in 15% ACN solution containing 0.05% TFA and passed through 0.45 μm syringe filters. Subsequently, were injected into the column and the elution was performed using buffer A (95% H_2_O + 5% ACN containing 0.05% TFA) and buffer B (100% ACN containing 0.05% TFA) with a gradient of 10–30%. The fractions were detected at 220 nm. Fractions were collected manually and lyophilized for further experiments.

#### Mass spectroscopy

The fractions obtained by RP-HPLC were subsequently examined by tandem mass spectrometry to identify the peptides responsible for the biological activities. Samples were first dissolved in 5% ACN containing 0.1% TFA and subsequently mixed with an equal volume of saturated α-cyano-4-hydroxycinnamic acid (CHCA). A volume of 1 μL of the resultant mixture was spotted on the MALDI plate and air dried for 10 min. The samples were then analyzed using a Autoflex Speed MALDI-TOF/TOF (Bruker Corporation, Billerica, MA, USA) in positive reflectron mode in a range from 500 to 3000 Da, and a voltage of 20 kV. Tandem mass spectra were analyzed by Peak Studio v7.5 (Bioinformatics Solutions, Waterloo, Canada), in combination with automated de novo sequencing using PEAK DB search engines. The mass error tolerance was set to 0.1 Da for all spectra. The sequences were also manually confirmed with the software mMass v5.5.0 (http://www.mmass.org).

### Peptide synthesis

P1 (GLPGAAGP), P2 (GRDGEP), and P3 (MTGTQGEAGR) were synthesized by PurePep Chorus, (Gyros Protein Technologies, Uppsala, Sweden) with a Rink amide 4-methylbenzhydrylamine (MBHA) resin (0.36 mmol/g) alongside with HBTU and DIEA as coupling reagents. The cleavage was performed under TFA/water/thioanisol/ethanedithiol (87.5/5/5/2.5, v/v/v/v).

### Biological assessment of the synthesized peptides

The synthesized peptides were investigated for ACE and DPP-IV inhibitory activity, and antibacterial potential against *E. coli, P. aeruginosa, S. aureus,* and *K. pneumoniae*. Their cytotoxicity was also analyzed using the method described by Benjakul et al.^20^, with some modifications. Briefly, Caco-2 and L929 cell lines were cultured in Dulbecco's Modified Eagle Medium and the RAW264.7 cell line in RPMI 1640 medium and incubated at 5% CO_2_ and 37 °C. All media were supplemented with 10% fetal bovine serum. Each cell line (100 µL at 2 × 10^4^ cells mL^−1^) was seeded onto 96-well microplates and mixed with an equal volume of each peptide at 1, 2, 4, and 6 mg mL^−1^. Subsequently, the microplate was incubated for 72 h, followed by a cell viability assay using the MTT I proliferation kit (Roche Diagnostics, Basel, Switzerland). The absorbance was recorded at 570 nm.

### Bioinformatics

The molecular structures of the synthesized peptides were obtained and optimized using ChemOffice Professional Software v17.1 (PerkinElmer, Waltham, MA, USA). The crystal structures of human ACE (PDB ID: 1o8A) and human DPP-IV (PDB ID: 5j3j), retrieved from the Protein Data Bank (www.rcsb.org) were used as target receptors for the peptides. AutoDock Vina software was used for the bioinformatics process due to its adaptability^21^. Afterward, simulations were performed to evaluate the docking response of the peptides onto the rigid active site of the receptors, and the docked molecules were automatically ranked according to their calculated binding affinities.

### Biostatistics

The mean values of the specimens were analyzed by SPSS Statistics v25 (IBM Corp., Armonk, NY, USA). The mean values were compared using one-way analysis of variance (ANOVA) and Tukey’s test. *P*-value < 0.05 was considered statistically significant. All data presented were obtained from three independent replicates.

## Results

### Hydrolysis and depolymerization

The results of 6 h of hydrolysis reaction are shown in Table 1. The DP was found to be not linearly related to the hydrolysis time, instead increasing as the hydrolysis time increased. The first 3 h of reaction caused more than 60% of the total hydrolysis, with the DP reaching 20.03%. Afterward, the DP continued to slowly, but significantly ascend. The final DP of 33.34% was achieved at 6 h of hydrolysis. As the results indicated, the ACE and DPP-IV inhibition by 6 h hydrolysates, at the concentration of 1 mg mL^−1^, were of 87.91% and 67.33%, respectively. These values were significantly higher than those from hydrolysates obtained with lower hydrolysis time. The antibacterial activity test also showed that the MIC of the hydrolysates against *E. coli* was the lowest (2.66 mg mL^−1^) at 6 h of hydrolysis. According to these results, there was a direct relationship between the DP value and ACE and DPP-IV inhibition, as well as with antibacterial activity. Due to the significantly higher biological activity of the hydrolysates obtained upon 6 h of hydrolysis, these were chosen for further experiments.

**Table 1:** Biological activities of gelatin hydrolysates (1 mg mL^-1^).

Hydrolysis time (h)	DP (%)	ACE inhibition (%)	DPP-IV inhibition (%)	MIC for *E. coli* (mg mL^−1^)
1	9.25 ± 0.02^e^	27.33 ± 0.11f.	22.68 ± 0.01f.	N/A
2	15.12 ± 0.01^d^	34.85 ± 0.10^e^	31.46 ± 0.02^e^	8.66 ± 0.66^a^
3	20.03 ± 0.03^c^	40.40 ± 0.10^d^	35.09 ± 0.04^d^	7.33 ± 0.66^b^
4	29.88 ± 0.02^b^	56.74 ± 0.09^c^	45.50 ± 0.02^c^	6.00 ± 0.00^c^
5	33.22 ± 0.03^a^	71.08 ± 0.07^b^	52.99 ± 0.06^b^	4.00 ± 0.00^d^
6	33.34 ± 0.02^a^	87.91 ± 0.05^a^	67.33 ± 0.31^a^	2.66 ± 0.66^e^

Data are shown as mean ± standard error of three replicates. Different superscript letters in the same column represent the significant difference (*P* < 0.05).

ACE, angiotensin-converting enzyme; DP, degree of depolymerization; DPP-IV, dipeptidyl peptidase IV; MIC, minimum inhibitory concentration; N/A, mean not applicable.

### Peptidomics approach

#### Ultrafiltration

After passing the hydrolysates through ultrafiltration units, the resultant fragments were analyzed for antihypertensive, antidiabetic, and antibacterial activities (Table 2). The results revealed that the fragments with molecular weight below 3 kDa were the most active. Their ACE inhibitory activity showed the lowest IC_50_ (0.20 ± 0.05 mg mL^−1^), which was significantly lower than those of fractions with higher molecular weights. Similarly, this fraction also showed the highest DPP-IV inhibitory activity, with an IC_50_ of 0.52 ± 0.02 mg mL^−1^. The MIC of the fraction < 3 kDa (≤ 0.50 ± 0.00 mg mL^−1^) was significantly lower than that of other fractions. Therefore, the fraction < 3 kDa was freeze dried and kept at − 20 °C for further purification.

**Table 2:** Biological activities of gelatin hydrolysate fractions obtained by ultrafiltration separation.

Fraction	ACE IC_50_ (mg mL^−1^)	DPP-IV IC_50_ (mg mL^−1^)	MIC for *E. coli* (mg mL^−1^)
F < 3	0.20 ± 0.05^c^	0.52 ± 0.02^c^	≤ 0.50 ± 0.00^c^
3 < F < 10	1.20 ± 0.02^b^	1.33 ± 0.02^b^	4.00 ± 0.00^b^
10 < F < 30	2.78 ± 0.05^a^	2.64 ± 0.05^a^	8.66 ± 0.66^a^

Data are shown as mean ± standard error of three replicates. Different superscript letters in the same column represent significant differences (*P* < 0.05).

ACE, angiotensin-converting enzyme; DPP-IV, dipeptidyl peptidase IV; IC_50_, half maximal inhibitory concentration; MIC, minimum inhibitory concentration.

#### Ion exchange chromatography

**Figure 1:**
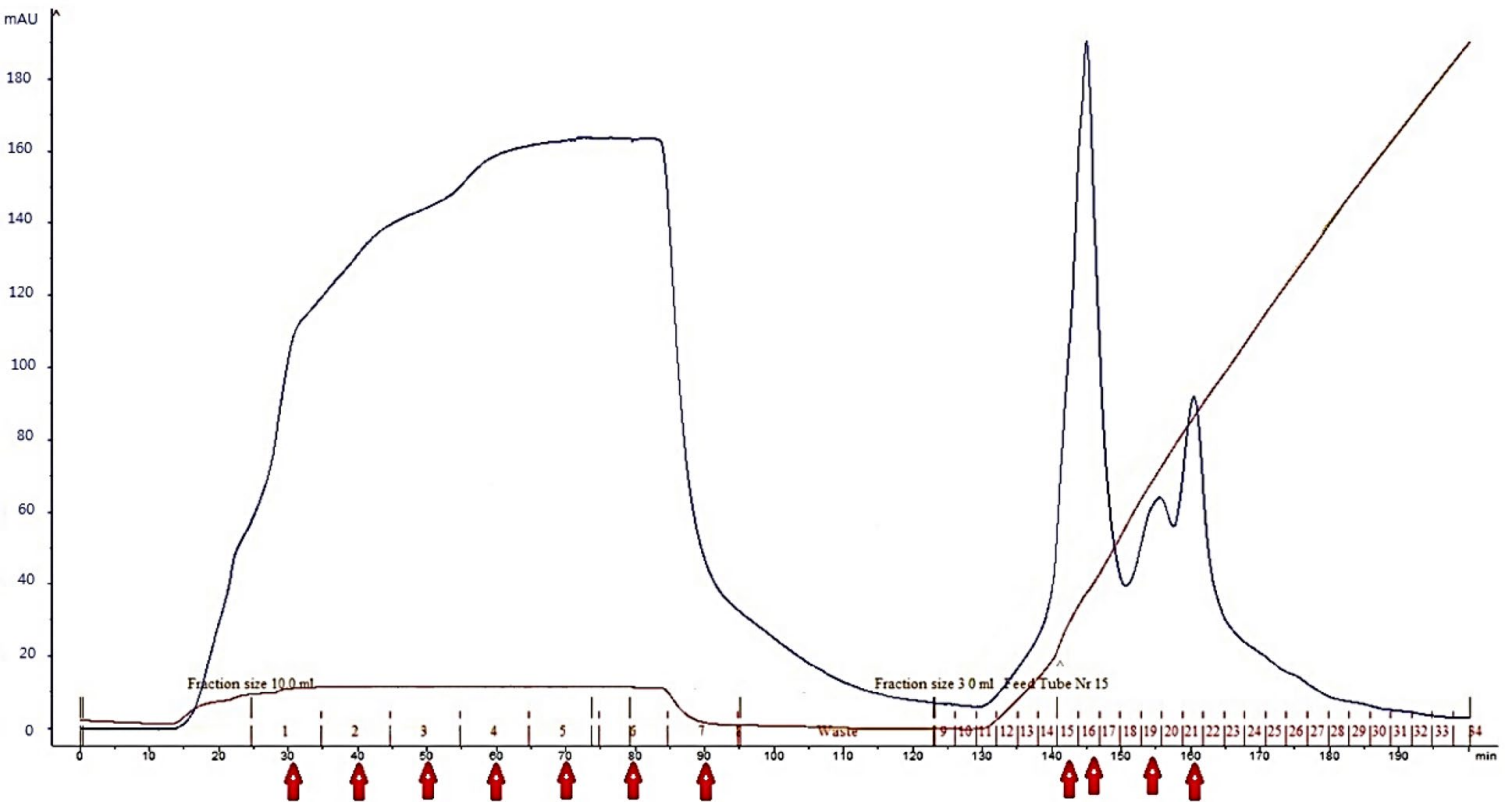
Ion exchange chromatogram obtained from the fraction < 3 kDa using a Q column.

The ion exchange chromatography was performed to purify the fraction < 3 kDa (Fig. 1), which the respective chromatogram showed 11 active fractions (Table 3). Detailed analysis of their biological activities revealed that the fraction 16 had the highest ACE and DPP-IV inhibitory activities (IC_50_ of 0.76 ± 0.00 and 0.85 ± 0.02 mg mL^−1^, respectively), whereas the fraction 15 presented the highest antibacterial activity (MIC = 0.5 mg mL^−1^). However, due to the low and in some cases lack of significance differences between the fractions 15, 16, 19, and 21, they were pooled and lyophilized for further purification.

**Table 3:** Biological activities of gelatin hydrolysate fractions obtained by anion exchange separation.

Fraction	ACE IC_50_ (mg mL^−1^)	DPP-IV IC_50_ (mg mL^−1^)	MIC for *E. coli* (mg mL^−1^)
1	10.52 ± 0.26^a^	9.45 ± 0.02^a^	N/A
2	9.95 ± 0.07^a,b^	9.17 ± 0.02^b^	N/A
3	9.64 ± 0.22^b^	8.25 ± 0.03^e^	N/A
4	8.73 ± 0.17^c^	9.14 ± 0.01^b^	N/A
5	10.10 ± 0.23^a,b^	8.95 ± 0.02^c^	N/A
6	8.82 ± 0.07^c^	8.40 ± 0.02^d^	N/A
7	10.48 ± 0.00^a^	N/A	N/A
15	1.00 ± 0.01^d^	1.05 ± 0.03f.	0.50 ± 0.00^c^
16	0.76 ± 0.00^d^	0.85 ± 0.02^ g^	1.00 ± 0.00^b^
19	0.98 ± 0.01^d^	1.06 ± 0.00f.	1.33 ± 0.33^a^
21	0.93 ± 0.02^d^	0.98 ± 0.00f.	1.33 ± 0.33^a^

Data are shown as mean ± standard error of three replicates. Different superscript letters in the same column represent significant differences (P < 0.05).

ACE, angiotensin-converting enzyme; DPP-IV, dipeptidyl peptidase IV; IC_50_, half maximal inhibitory concentration; MIC, minimum inhibitory concentration; N/A, means not applicable.

#### SPE

The next fractionation step was performed by SPE with a C18 column to enhance the purity of the sample. As shown in Table 4, the samples showed a positive relationship between their biological activities including ACE and DPP-IV inhibition, antibacterial activity, and ACN concentration. The highest ACE and DPP-IV inhibitory potential (IC_50_ = 0.78 and 0.82 mg mL^−1^, respectively), and antibacterial activity against *E. coli* (MIC = 0.5 mg mL^−1^) were related to the fraction obtained by 30% ACN, whereas the lowest ACE and DPP-IV inhibition (IC_50_ = 1.08 and 1.15 mg mL^−1^), and antibacterial activity (MIC = 1.33 mg mL^−1^) were related to 5% ACN sample.

**Table 4:** Biological activities of gelatin hydrolysate fractions obtained by solid phase extraction.

ACN fraction	ACE IC_50_ (mg mL^−1^)	DPP-IV IC_50_ (mg mL^−1^)	MIC for *E. coli* (mg mL^−1^)
5	1.08 ± 0.12^a^	1.15 ± 0.03^a^	1.33 ± 0.33^c^
10	0.97 ± 0.03^b^	1.02 ± 0.01^b^	1.00 ± 0.00^b^
15	0.87 ± 0.01^c^	0.94 ± 0.02^c^	1.00 ± 0.00^b^
20	0.84 ± 0.00^c^	0.91 ± 0.00^c^	1.00 ± 0.00^b^
25	0.81 ± 0.03^c,d^	0.86 ± 0.04^d^	0.50 ± 0.00^c^
30	0.78 ± 0.00^d^	0.82 ± 0.01^d^	0.50 ± 0.00^c^

Data are shown as mean ± standard error of three replicates. Different superscript letters in the same column represent significant differences (*P* < 0.05).

ACE, angiotensin-converting enzyme; ACN, acetonitrile; DPP-IV, dipeptidyl peptidase IV; IC_50_, half maximal inhibitory concentration; MIC, minimum inhibitory concentration.

#### RP-HPLC

**Figure 2:**
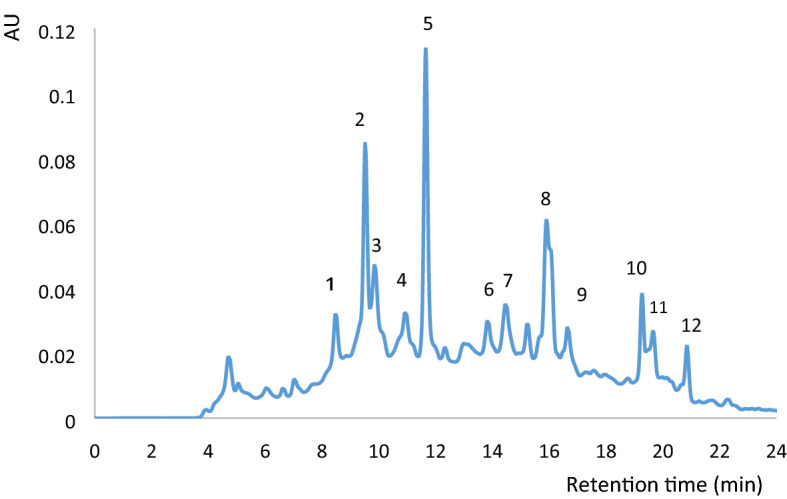
Reverse-phase high-performance liquid chromatogram obtained from the fraction < 3 kDa.

The purification of hydrolysates by RP-HPLC revealed 12 fractions (Fig. 2), among which the fractions 5 and 8–12 were the most active (Table 5). Regarding ACE and DPP-IV inhibitory activities, the IC_50_ values of fraction 5 (0.94 and 0.90 mg mL^−1^, respectively) and fraction 12 (0.97 and 0.99 mg mL^−1^, respectively) were significantly higher than those of the other fractions. Moreover, the MIC values of fractions 5 and 12 were 2.00 and 1.00 mg mL^−1^, respectively. The fraction 8, which had the highest absorbance in the chromatogram, showed the highest antibacterial activity (MIC ≤ 0.50 mg mL^−1^, while the ACE and DPP-IV inhibitory activities were 1.10 and 1.35 mg mL^−1^, respectively.

**Table 5:** Biological activities of gelatin hydrolysate fractions obtained by reverse-phase high-performance liquid chromatography.

Fraction	ACE IC_50_ (mg mL^−1^)	DPP-IV IC_50_ (mg mL^−1^)	MIC for *E. coli* (mg mL^−1^)
1	8.64 ± 0.11^a,b^	N/A*	N/A
2	7.16 ± 0.43^c,d^	8.51 ± 0.09^a^	N/A
3	8.55 ± 0.42^a,b^	N/A	N/A
4	9.03 ± 0.94^a^	N/A	N/A
5	0.94 ± 0.93f.	0.90 ± 0.24^c^	2.00 ± 0.00^a^
6	7.95 ± 0.52^b,c^	8.36 ± 0.10^a^	N/A
7	6.83 ± 0.37^d^	N/A	N/A
8	1.10 ± 0.19^e^	1.35 ± 0.00^b^	≤ 0.50 ± 0.00^d^
9	1.21 ± 0.72^e^	1.67 ± 0.07^b^	1.66 ± 0.66^b^
10	1.05 ± 0.24^e^	1.31 ± 0.19^b^	1.33 ± 0.33^b^
11	1.00 ± 0.87^e^	1.02 ± 0.44^b,c^	2.00 ± 0.00^a^
12	0.97 ± 0.44^e,f^	0.99 ± 0.12^c^	1.00 ± 0.00^c^

Data are shown as mean ± standard error of three replicates. Different superscript letters in the same column represent significant differences (*P* < 0.05).

ACE, angiotensin-converting enzyme; DPP-IV, dipeptidyl peptidase IV; IC_50_, half maximal inhibitory concentration; MIC, minimum inhibitory concentration; N/A, means not applicable.

#### Mass spectroscopy and peptide synthesis

After fractionation of the hydrolysates by RP-HPLC, the most active fraction was examined by tandem mass spectrometry to identify specific peptide sequences. After fragmentation into y and b ions, the peptides responsible for the observed biological and functional activities were identified as GPLGAAGP (P1), GRDGEP (P2), and MTGTQGEAGR (P3) (Fig. S1). Therefore, all three peptides (P1, P2, and P3) were synthesized and purified by RP-HPLC for further analysis, including their antibacterial activity against four pathogenic microorganisms (*E. coli*, *P. aeruginosa*, *S. aureus*, and *K. pneumoniae*), as well as bioinformatics studies to confirm the in vitro results.

### Biological assessment of synthesized peptides

#### Antibacterial activity

**Figure 3:**
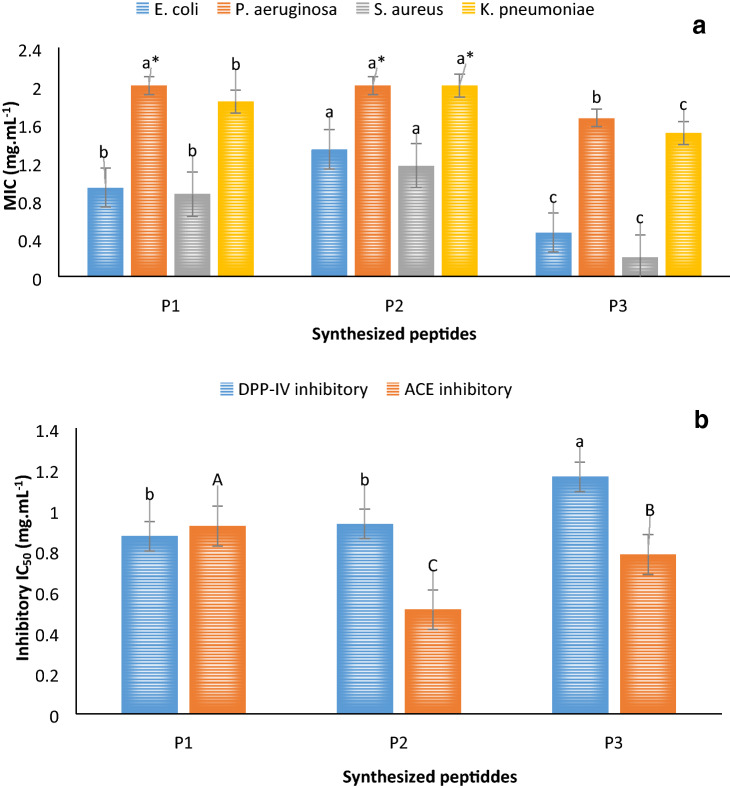
Biological assessment of the synthesized peptides. (**a**) Antibacterial activity, and (**b**) ACE and DPP-IV inhibition of the three peptides (P1, P2, and P3). *Indicates minimum inhibitory concentration values of ≥ 2 mg mL^−1^. ACE, angiotensin-converting enzyme; DPP-IV, dipeptidyl peptidase IV; IC_50_, half maximal inhibitory concentration.

The antibacterial activities of P3 and P2 against all strains were found to be the highest and the lowest, respectively (Fig. 3a). The MIC values of P3 against *E. coli*, *P. aeruginosa*, *S. aureus*, and *K. pneumoniae* were 0.46, 1.66, 0.20, and 1.50 mg mL^-1^, respectively, whereas those of P2 were 1.33, ≥ 2.00, 1.16, and ≥ 2.00 mg mL^−1^, respectively. P1 showed the lowest activity against all strains with MIC values of 0.93, ≥ 2.00, 0.86, and 1.83 mg mL^−1^. Overall, the effect of P3 was more marked against *S. aureus*, similar to P1 and P2, whereas the lowest activity of all peptides was observed against *P. aeruginosa*. For more investigations, the antibacterial activity of the mixture of the three peptides (1:1:1) was examined. The combined antibacterial activity against *E. coli*, *P. aeruginosa*, *S. aureus*, and *K. pneumoniae* was 0.86, 1.33, 0.2, and 1.66 mg mL^−1^, respectively. Altogether, these results showed that the activity of the peptides was lower against gram-negative bacteria; however, in comparison with previous data, the activity of these peptides was strong enough to be considered as antibacterial agents against both gram-negative and -positive pathogens.

Given that the peptide charge can affect its antibacterial activity, the peptides were investigated based on their net charge at pH 7 using a peptide calculator database (https://pepcalc.com/). P1 and P3 showed no charge, whereas the charge of the P2 was − 1. The hydropathy results revealed that P2 had more charged amino acids and the charge state of the peptide was positive (+ 1) at pH values lower than 4. The charge state of P3 was positive (+ 1) at pH values lower than 5, whereas from pH 5 to 8 it was neutral. P1 showed a neutral state until pH 9 and after that decreases to − 1 charge.

The peptides were also used as input data sequences to the online server database (http://aps.unmc.edu/AP/prediction/prediction_main.php) to predict their protein interaction index and their ability of acting as antibacterial agents. The results showed that the protein interaction index for P1, P3, and P2 was − 1.42, 2.54, and 4.76 kcal∙mol^−1^, respectively.

#### Antihypertensive and antidiabetic activity and cytotoxicity of the peptides

The antihypertensive (ACE inhibition) and antidiabetic (DPP-IV inhibition) activities of the identified peptides are depicted in Fig. 3b. The results indicated that the ACE IC_50_ value of P2 (0.51 mg mL^−1^) was significantly higher than that of P1 (0.92 mg mL^−1^) and P3 (0.78 mg mL^−1^). The mixture of the three peptides (1:1:1) showed an IC_50_ of 0.83 mg mL^−1^ for ACE inhibition.

Similar results were observed for DPP-IV inhibition, with P3 showing the lowest inhibitory potential (IC_50_ = 1.16 mg mL^−1^), whereas P2 (IC_50_ = 0.93 mg mL^−1^) and P1 (IC_50_ = 0.87 mg mL^−1^) did not showed a significant (*P* < 0.05) difference among them. The mixture of three peptides (1:1:1) showed an IC_50_ of 1.15 mg mL^−1^ for DPP-IV inhibition.

These results were then validated by bioinformatics tools and the binding energy of the interaction between each peptide with each enzyme was calculated.

**Figure 4:**
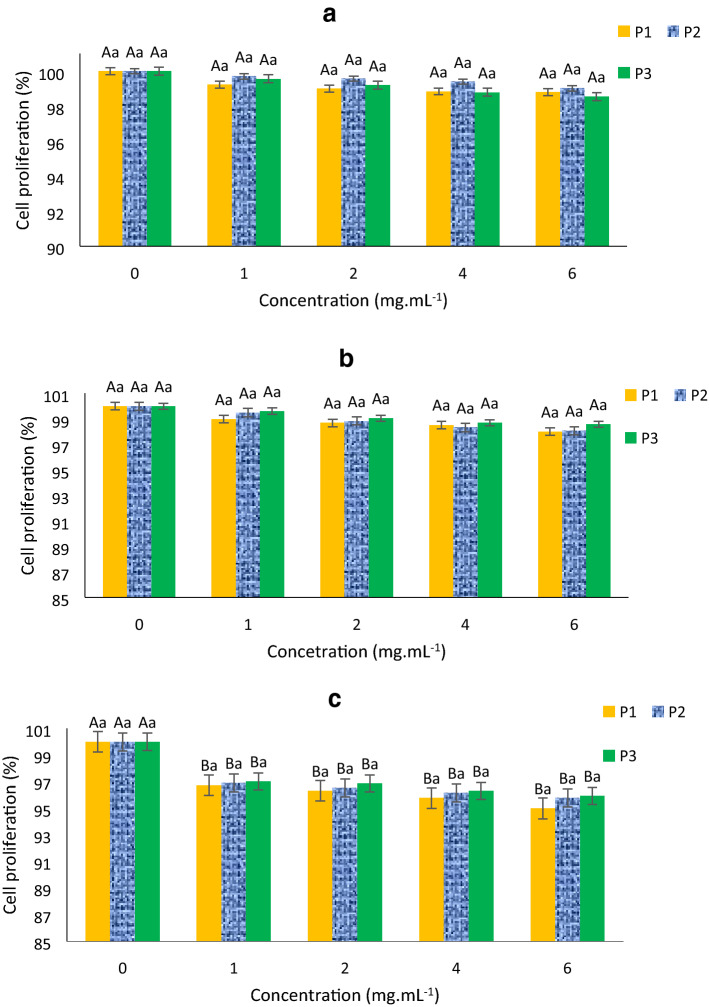
Cytotoxicity of the synthesized peptides at different concentrations. The cytotoxicity of the three peptides (P1, P2, and P3) were tested by the inhibition of the proliferation of (**a**) L929, (**b**) RAW264.7, and (**c**) Caco-2 cell lines. Different uppercase and lowercase letters represent the significant difference among various concentrations of the same peptide and various peptides at the same concentrations, respectively.

Cytotoxicity assays indicated that proliferation of the cell lines was mildly affect by the peptides compared with untreated cells (Fig. 4). The Caco-2 cell line was the most affected by different peptides, with cell proliferation rates of 95.00%, 95.77%, and 95.94% with P1, P2, and P3 at 6 mg mL^−1^, respectively. The other two cell lines had a proliferation rate above 98% for all three peptides. Nevertheless, no significant differences (*P* > 0.05) were observed between the tested cell lines treated with peptide concentrations ranging from 1 to 6 mg mL^−1^.

#### Bioinformatics

**Figure 5:**
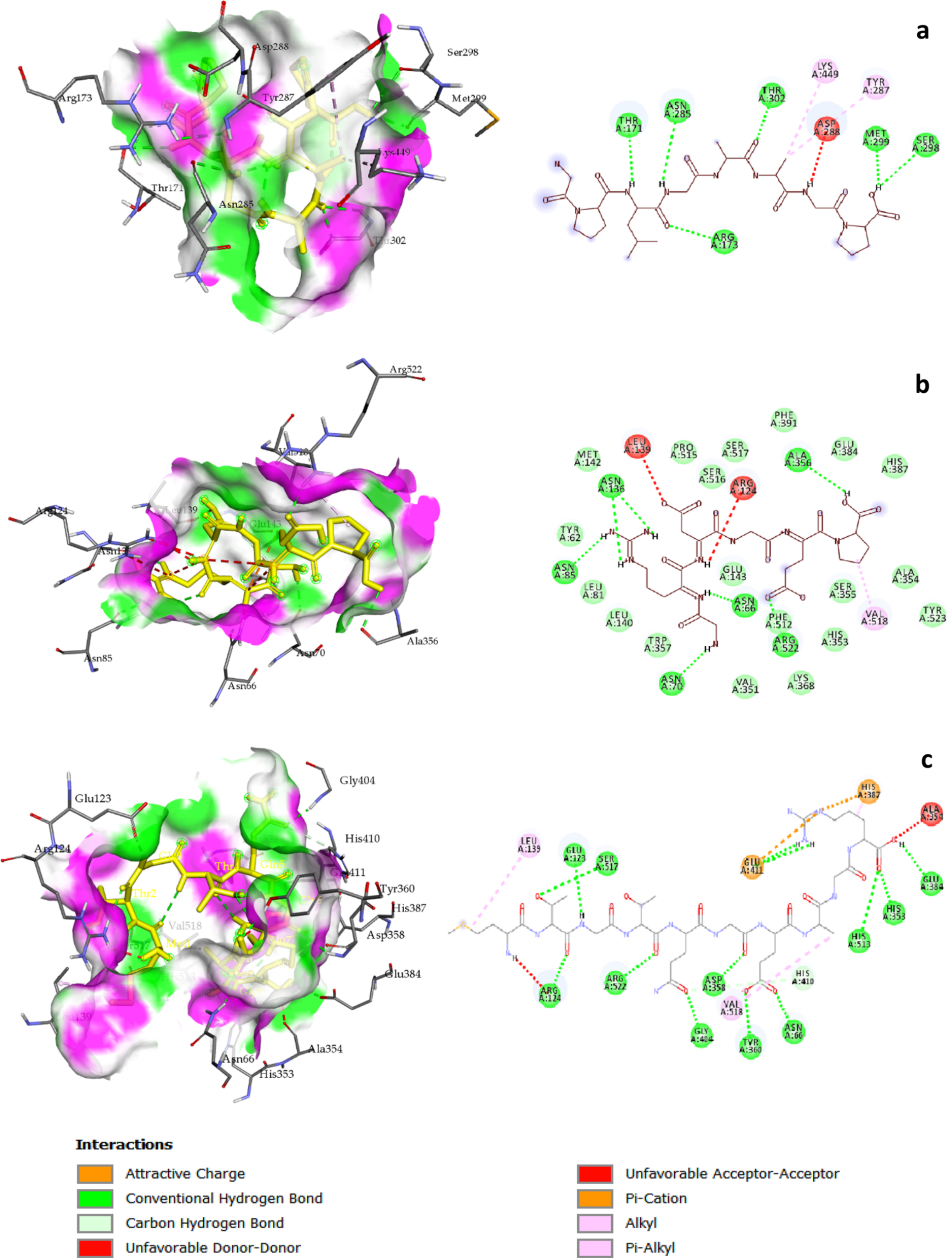
Computational simulation of the docked peptides on the human angiotensin-converting enzyme (ACE) structure with their interactions. (**a**) GPLGAAGP (P1), (**b**) GRDGEP (P2), and (**c**) MTGTQGEAGR (P3).

**Figure 6:**
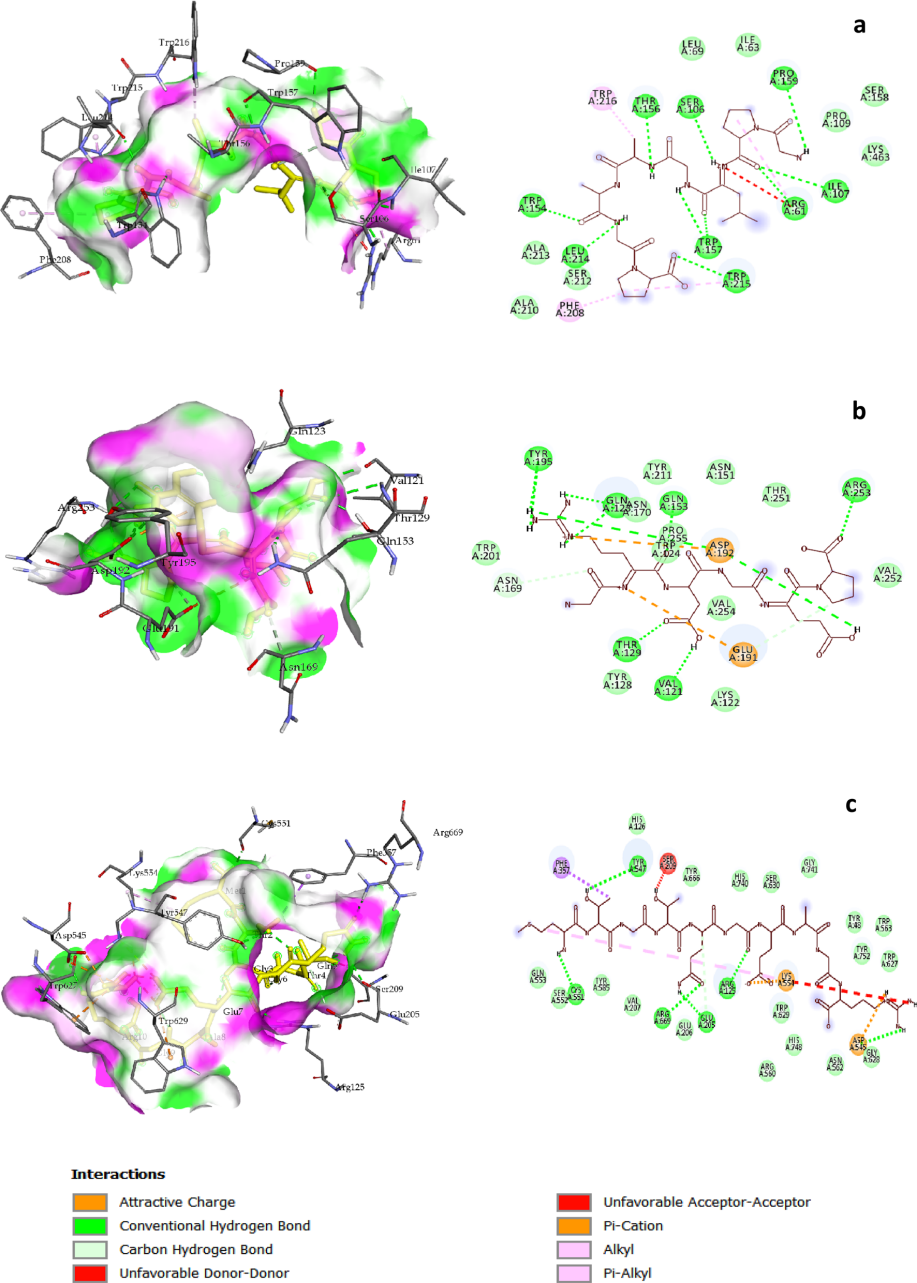
Computational simulation of the docked peptides on the human dipeptidyl peptidase IV (DPP-IV) structure with their interactions. (**a**) GPLGAAGP (P1), (**b**) GRDGEP (P2), and (**c**) MTGTQGEAGR (P3).

The in silico molecular docking data agreed with the obtained in vitro ACE and DPP-IV inhibitory assays. In the case of ACE (Fig. 5), the results showed that the binding affinity of P2 (8.1 kcal mol^−1^) was more negative than those of P1 (6.3 kcal∙mol^−1^) and P3 (7.4 kcal∙mol^−1^), suggesting that the ACE inhibitory activity of the peptides was P2 > P3 > P1. The in vitro biological assessment for three peptides showed the same tendency. In the case of DPP-IV (Fig. 6), the binding affinity of P1 (8.1 kcal mol^−1^) was more negative than those of P2 (7.3 kcal∙mol^−1^) and P3 (6.6 kcal∙mol^−1^). Therefore, the DPP-IV inhibitory activity of the peptides was as P1 > P2 > P3, which agreed with the in vitro results.

The binding energy of the interaction between each peptide with each enzyme was also calculated, showing that the root-mean-square deviation of atomic positions (RMSD) was of 0.000.

## Discussion

### DP

The DP of protein is an important factor for determining the functional characteristics of peptides. The present study showed that the addition of actinidin significantly increased the DP and improved the functionality of the hydrolysates. Nongonierma, et al.^22^ showed similar results for the DPP-IV inhibitory activity of peptides derived from milk protein, with the high DPP-IV inhibitory potential of H4 and H11 peptides (with a degree of hydrolysis of 11.27% and 12.19%, respectively) could be due to the long hydrolysis time (≥ 150 min). Another study conducted by Jamdar et al.^23^ showed that ACE inhibitory activity of peanut protein hydrolysates could be obtained by subtilisin A treatment, which was directly related to the DP. The effect of DP (50–100%) on the antibacterial activity of palm kernel expeller peptides was also investigated by Tan, et al.^24^. They demonstrated that 70% DP resulted in the highest antibacterial activity against gram-positive bacteria with an IC_50_ of 200 µg mL^−1^, whereas no or limited activity against *E. coli* and other gram-negative bacteria was observed. Taken together, these findings suggest that the herein generated hydrolysates represent a highly active source of multifunctional peptides. Therefore, the hydrolysates underwent further purification and analyses.

### Peptidomics approach

#### Ultrafiltration

The observed higher antibacterial activity after ultrafiltration can be explained by the higher concentration of lower molecular weight peptides in the solution. A previous study in which the peptic hydrolysate of Pacific cod skin was fractionated into different molecular weight solutions using ultrafiltration membranes of 10, 5, and 1 kDa^25^, revealed that among the obtained four fractions of > 10, 5–10, 1–5, and < 1 kDa peptides, the one of < 1 kDa hold the higher ACE inhibitory activity (70% at 0.5 mg mL^−1^). Another study performed by Sha et al.^26^ showed that the porcine skin gelatin hydrolysates with the molecular weight of < 1 kDa obtained from ultrafiltration, with the cutoff of 2.5 and 1 kDa, had the highest DPP-IV inhibitory activity. These results agree with the present findings, based on which the fraction < 3 kDa was chosen for further purification and analyses due to its higher biological activity than the other fractions.

#### Ion exchange chromatography

The observed reduction of the biological activity of the fractions after anion exchange separation could have resulted from the loss of synergistic peptides. A previous investigation performed on a fraction of higher molecular weight hydrolysates obtained from the same gelatin source and enzymatic reaction, also showed that the antioxidant and anticancer activities of the fraction were decreased after anion exchange chromatography (DEAE-Sephadex A-25 anion exchange column)^2^. Furthermore, another study performed to understand the effect of bioactive peptides from *Oreochromis niloticus* skin gelatin on ACE activity and radical scavenging^27^ demonstrated that the ion exchange chromatography dramatically reduced biological activities of the peptides due to the loss of some synergistic peptides cooperating with their ACE inhibitory and radical scavenging activities. Herein, based on the similar results among samples and enhanced biological activities, the fractions 15, 16, 19, and 21 were pooled and lyophilized for further assessment.

#### SPE

A positive relationship between the biological activities of the peptides and their ACN concentration was observed upon SPE; hence, their ACE and DPP-IV inhibitory potential, as well as antibacterial activity, could be related to the hydrophobicity of the fractions. Despite the structure–activity relationship of the peptides had not yet been fully elucidated, some structural characteristics of peptides previously determined were shown to impact on their biological activities. In this regard, Lacroix and Li-Chan^28^ showed that the presence of proline/hydroxyproline in peptide sequences is an important to have a good DPP-IV inhibitory activity. Another study revealed that ACE inhibitory activity is strongly affected by the C-terminal sequence, especially in hydrophobic amino acids, such as proline^29^. Additionally, the antibacterial activity of bioactive peptides is also affected by several structural parameters such as the number of α-helices, charge, chain length, and hydrophobicity^30^. Therefore, it is reasonable to assume that fraction with higher ACN concentrations may be more biologically active. However, owing to the low quantity of the recovered fraction of 30% (v/v) ACN after lyophilization and considering no statistical differences in biological activities were detected between these two fractions, this sample was pooled with the fraction of 25% (v/v) ACN.

#### RP-HPLC and mass spectroscopy

The fractionation by RP chromatography is a good procedure to purify peptides based on their relative hydrophobicity, as more hydrophobic peptides will be retained for longer. Moreover, longer retention time indicates higher amounts of hydrophobic or aromatic amino acid in the peptide structure^31^. In the present study the chromatogram of the isolated fractions only showed a good ACE inhibitory activity upon fraction 5, which agrees with the literature. In a research on peptides derived from hen egg white lysozyme, 12 fractions were detected after purification by RP-HPLC of which the most active fractions concerning antihypertensive activity were the fractions 2, 7, and 9 (IC_50_ = 0.01, 0.005, and 0.014 mg mL^−1^, respectively)^32^.

As it was mentioned before, ACE and DPP-IV inhibitory potential, and antibacterial activity of the extracts could be related to the hydrophobicity of the fractions; however, the amino acid composition and sequence of the peptides can also significantly alter their biological proprieties. Therefore, the amino acid sequence of the highly active fractions were evaluated next by MALDI-TOF/TOF mass spectroscopy. Three identified peptides were found to be rich in glycine and proline, which could contribute for their biological activity. Thus, the peptides were synthesized for a detailed analysis.

### Biological assessment of synthesized peptides

#### Antibacterial activity

The cell membrane composition could be a very important factor impacting on the action of antibacterial peptides. For example, the existence of an outer membrane, which consists of a lipopolysaccharide layer in gram-negative bacteria, may be the cause of lower antibacterial activity of peptides against them^33^. However, it has been suggested that the lipopolysaccharide binding ability of peptides is neither directly nor indirectly related to their antibacterial activity. Once the peptides passed through the broken outer membrane, the interaction with the cytoplasmic membrane is the key factor of their antibacterial efficiency, which could in turn be affected by their concentration and sequence^34^. The present findings are in agreement with those of a previous study, which demonstrated that the antibacterial effect of peptides from oyster (*Crassostrea gigas*) against *S. aureus* (with an IC_50_ of 18.6 ± 20 µg mL^−1^) was significantly stronger than that against other bacteria strains^35^.

The protein-binding potential (Boman index) is an important factor to determine the potential of protein interactions based on the peptide sequence. This index is calculated using the sum of free energies of the corresponding side chains for transfer from cyclohexane to water and divided by the total number of the amino acids. This important factor could be used to interpret the ability of a peptide to bind either the microbial cell membrane or other proteins as receptors. A Boman index higher than 2.48, indicates high binding potential^36^. Therefore, the present results suggest that the antibacterial mechanism of the P1 is not through the interaction with the cell membrane. In contrast, the P2 and P3 showed high Boman index, suggesting that they may interact with membrane portions as receptors. The online database also represents some antibacterial peptides rich in proline (just like P1 in the present study) and tryptophan. Regarding the antibacterial activity of the mixture of three peptides, it is possible to conclude that these peptides may have a synergistic effect on their actions against some particular pathogens such as *P. aeruginosa*. However, the highest activity against other pathogens was the same as mentioned above. Furthermore, we cannot conclude that mixing the peptides is a good strategy to enhance their antibacterial activity against all bacteria.

#### Antihypertensive and antidiabetic activity

ACE is responsible for the conversion of the inactive decapeptide (angiotensin I), by cleaving a dipeptide from the C-terminus, into a vasoconstriction octapeptide (angiotensin II), as well as by the inactivation of bradykinin, which is as a vasodilator in the kallikrein-kinin system^18^. Therefore, ACE inhibition leads to an antihypertensive effect. The results of the present study revealed that all three identified peptides had good ACE inhibitory activities (IC_50_ < 1 mg mL^−1^), with the most potent ACE inhibitor being P2 (IC_50_ = 0.51 mg mL^−1^). Certainly, the amino acid sequence of the peptides is one of the most important factors affecting their ACE inhibitory activity, which can be strongly affected by the C-terminal sequence, especially by hydrophobic amino acids such as proline^29^. Indeed, the C-terminal amino acid in P1 and P2 was proline. However, the effect of the peptide structures on ACE inhibitory potential is not fully known. The identified P3 was found to have methionine and arginine at the N- and C-terminus, respectively, but its ACE inhibitory activity was significantly higher than that of P1 with a proline at the C-terminus. In a hexapeptide (VLAQYK) sequenced from beef hydrolysates, despite the presence of aliphatic amino acids at the N- and C-terminus instead of a proline residue, its ACE inhibition was of 30.1%^37^.

DPP-IV is an enzyme that participated in the incretin hormone system; thus, its inhibition can significantly decrease diabetes mellitus type II^4,5^. However, synthetic DPP-IV inhibitors have undesired side effects, such as hypoglycemia, weight gain, diarrhea, nausea, and abdominal pain^6^. Dipeptides with DPP-IV inhibitory IC_50_ values less than 100 mM usually have tryptophan/threonine/methionine at their N-terminus^38^, which is in agreement with the sequence of the herein identified P3. As it is mentioned previously, hydrophobic amino acids including alanine, glycine, isoleucine, leucine, phenylalanine, proline, methionine, tryptophan, and valine are responsible for the DPP-IV inhibitory activity of peptides, as these hydrophobic amino acids may facilitate the interaction of peptides with the enzyme active site. Hydrophobic pockets are believed to have an important effect on DPP-IV inhibition by peptides; however, their mechanism of action remains unknown^3,38^. Zhang et al.^39^ showed that the dodecapeptide SPTVMFPPQSVL, containing a proline at the second position of the N-terminus, and the octapeptide MHQPPQPL, containing a proline at the second position of the C-terminus, had moderate DPP-IV inhibitory activities. The researchers suggested that not only the peptide sequence, but also its length could affect the DPP-IV-inhibition mechanism. Analysis of the structure–function relationship of various DPP-IV-inhibitory peptides has suggested that the binding to DPP-IV is strongly influenced by the N-terminal amino acid^40^. Altogether, these findings indicated that a proline located in the N- or C-terminus of peptides, as well as in the penultimate positions, is an important factor regulating its biological activity^38–40^. Of note, the mixture of the peptides showed slightly higher ACE and DPP-IV inhibitory activity than P3 and P1 alone, respectively. However, this result should not encourage the development of the mixture as an opponent to the single peptides since P1 and P2 showed significantly higher ACE and DPP-IV inhibitory activities, respectively.

#### Cytotoxicity

Bioactive peptides obtained from gelatin hydrolysis are recognized as safe food ingredients with minimal adverse effects^20,41,42^. Previous studies, both in vitro and in silico^15,16^, demonstrated that fractions with different molecular weights obtained by ultrafiltration had no toxicity, further supporting the present findings. Nonetheless, to the best of our knowledge, only few studies evaluated the cytotoxicity of fish gelatin hydrolysates in comparison to normal cells. The cytotoxicity of collagen hydrolysates obtained from cartilage was monitored by Schauss, et al.^43^ as acute oral toxicity in Sprague Dawley rats. This study revealed that the 50% lethal dose was greater than 5000 mg kg^−1^ body weight. Benjakul et al. also showed that hydrolyzed collagen obtained from seabass skin in Wistar rat was safe, with the 50% lethal dose being higher than 5000 mg kg^−1^ body weight^20^. Based on this evidence as well as the present results, it is reasonable to consider that the peptides identified herein are safe for consumption due to their lack of cytotoxicity at different concentrations.

#### Bioinformatics

The molecular structures of the three peptides were simulated and energy minimized using ChemOffice, and they were docked onto the rigid structure of human ACE and DPP-IV. To confirm the validity of the docking process, the ACE and DPP-IV were separated from their specific ligands (Lisinopril and HL1, respectively) and re-docked onto the rigid structures of the corresponding apoenzymes.

The good binding affinity (more negative) of the synthesized peptides observed for docking onto ACE and DPP-IV pockets could be due to the large surface of the binding interactions and consequent high number of hydrogen bonds formed. The in silico results further confirmed the collected in vitro data. For ACE docking, the highest (6.3 kcal mol^−1^) and the lowest (8.1 kcal mol^−1^) binding energies were related to P1 and P2, respectively, which agreed with the ACE inhibitory results. In turn, for DPP-IV docking, the highest (6.6 kcal mol^−1^) and the lowest (8.1 kcal mol^−1^) binding energies were related to P3 and P1, respectively, which also confirmed the DPP-IV inhibitory results.

A study performed to identify ACE inhibitory peptides from *O. niloticus* skin gelatin, two peptides—GPEGPAGAR and GETGPAGPAGAAGPAGPR—were docked onto the rigid pocket of ACE^27^. The researchers stated that the binding affinity of these peptides was − 9.5 and − 9.3, respectively. However, it is noteworthy that several studies target the rabbit lung ACE, whereas the human ACE was used in the present study.

## Conclusions

In recent years, natural products have gathered increasing attention as sources of bioactive compounds due to their general safe profile. Moreover, the negative side effects of synthetic drugs have led consumers to consider natural medicinal compounds. Hypertensive and diabetes mellitus type II two of the most common chronic diseases worldwide. Hence, natural compounds with antimicrobial activity, as well as antihypertensive and antidiabetic properties, are currently the main goal of many investigations. This study aimed to discover multifunctional peptides from natural fish skin gelatin using enzymatic hydrolysis. After some purification steps, three multifunctional peptides were identified and their biological activity was confirmed by in vitro and in silico approaches. Additional studies are warranted to further explore the structure–function relationship of these peptides, which could pave the way for the development of more efficient and safer therapeutic agents.

## Supplementary Information

Supplementary Figure S1.
