# Supplementary material for: Structure–function engineering of novel fish gelatin-derived multifunctional peptides using high-resolution peptidomics and bioinformatics
Source: Sci Rep. 2021 Apr 1;11:7401. doi: 10.1038/s41598-021-86808-9 (PMC8016831; doi:10.1038/s41598-021-86808-9)
Supplement: Supplementary file 1 — Supplementary Figure S1. [file 41598_2021_86808_MOESM1_ESM.docx]

**Title:** Structure-function engineering of novel multifunctional peptides: High resolution peptidomics and bioinformatics approaches

**Running title:** Structure-function engineering of novel multifunctional peptides

Armin Mirzapour-Kouhdasht^a,b,d*^, Marzieh Moosavi-Nasab^a,b*^, Chul Won Lee^c^, Hyosuk Yun^c^, Jong-Bang Eun^d*^

^a^ Department of Food Science and Technology, School of Agriculture, Shiraz University, Shiraz, Iran

^b^ Seafood Processing Research Group, School of Agriculture, Shiraz University, Shiraz, Iran

^c^ Department of Chemistry, Chonnam National University, Gwangju, 61186, South Korea

^d^ Department of Integrative Food, Bioscience and Biotechnology, Chonnam National University, Gwangju, South Korea

***Corresponding Authors Email:**

[armin.mirzapour@shirazu.ac.ir](mailto:armin.mirzapour@shirazu.ac.ir) (A. Mirzapour-Kouhdasht)

[marzieh.moosavi-nasab@mail.mcgill.ca](mailto:marzieh.moosavi-nasab@mail.mcgill.ca) (M. Moosavi-Nasab)

[jbeun@jnu.ac.kr](mailto:jbeun@jnu.ac.kr) (J.B. Eun)


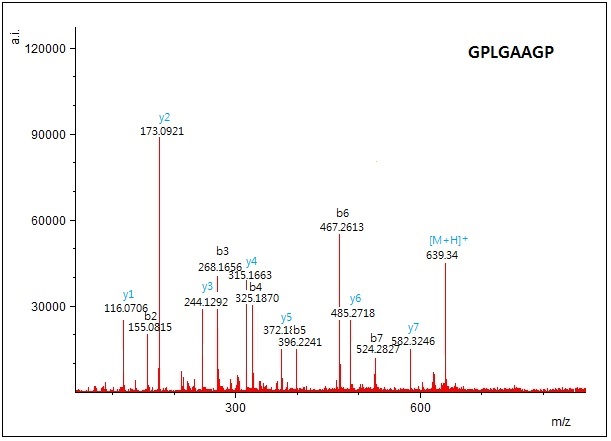


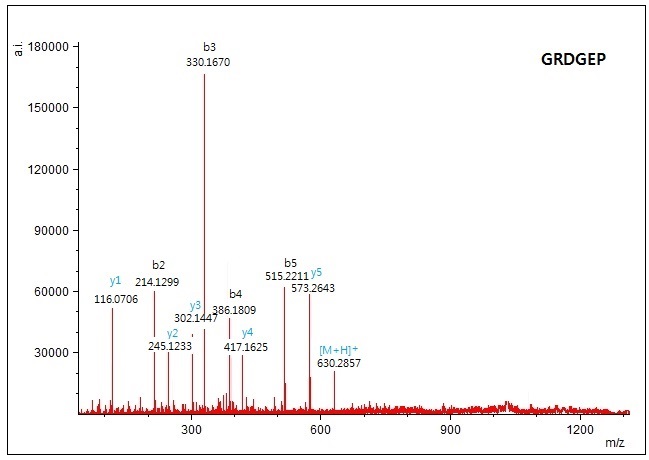


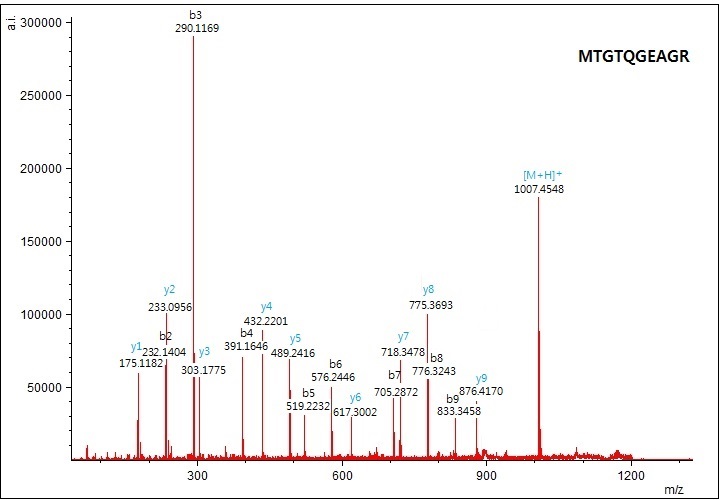


Fig S1. MALDI-TOF/TOF spectra of three identified peptides obtained from F<3kDa
